# The Toothbrush Microbiome: Impact of User Age, Period of Use and Bristle Material on the Microbial Communities of Toothbrushes

**DOI:** 10.3390/microorganisms8091379

**Published:** 2020-09-09

**Authors:** Marc-Kevin Zinn, Laura Schages, Dirk Bockmühl

**Affiliations:** Faculty of Life Sciences, Rhine-Waal University of Applied Sciences, 47533 Kleve, Germany; marc-kevin.zinn@hochschule-rhein-waal.de (M.-K.Z.); laura.schages@hsrw.eu (L.S.)

**Keywords:** toothbrush, oral microbiome, microbial contamination, antibiotic resistance

## Abstract

Toothbrushes play a central role in oral hygiene and must be considered one of the most common articles of daily use. We analysed the bacterial colonization of used toothbrushes by next generation sequencing (NGS) and by cultivation on different media. Furthermore, we determined the occurrence of antibiotic resistance genes (ARGs) and the impact of different bristle materials on microbial growth and survival. NGS data revealed that *Enterobacteriaceae*, *Micrococcaceae*, *Actinomycetaceae*, and *Streptococcaceae* comprise major parts of the toothbrush microbiome. The composition of the microbiome differed depending on the period of use or user age. While higher fractions of *Actinomycetales*, *Lactobacillales*, and *Enterobacterales* were found after shorter periods, *Micrococcales* dominated on both toothbrushes used for more than four weeks and on toothbrushes of older users, while in-vitro tests revealed increasing counts of *Micrococcus* on all bristle materials as well. Compared to other environments, we found a rather low frequency of ARGs. We determined bacterial counts between 1.42 × 10^6^ and 1.19 × 10^7^ cfu/toothbrush on used toothbrushes and no significant effect of different bristles materials on bacterial survival or growth. Our study illustrates that toothbrushes harbor various microorganisms and that both period of use and user age might affect the microbial composition.

## 1. Introduction

Toothbrushes play an essential role in oral hygiene and thus might be a potential reservoir of pathogens in patients with oral diseases as well as healthy adults [1,2], posing a possible risk of infection due to recontamination of the oral cavity. A contamination might be caused by microorganisms originating from the oral cavity, the environment, the skin microbiome or aerosols from toilet flushing as well as from storage containers [3,4,5]. Besides, bacteria are able to attach to toothbrush bristles where they survive or even propagate, which may result in a transmission to the user and possibly (re-)infections [6].

Since microbial cells can remain viable on toothbrushes, a transmission to the user or between household members during storage [7] can be assumed for a period of several days [8], indicating that the use of contaminated toothbrushes can contribute to the spread of microorganisms in the oral cavity [3]. Thus, toothbrushes must be considered a source of potential pathogens due to attachment of various types of bacteria [5,9,10,11,12,13], viruses [8], and fungi [14,15] when used and stored under normal conditions. Even after a use phase of only 24 h, toothbrushes have been shown to be extensively contaminated with *Streptococcus mutans* [16], and there is good evidence that the microbial load increases with an increasing period of usage [14,17,18]. Higher amounts of pathogens on toothbrushes might especially pose a health risk for risk groups including infants, elderly people, pregnant women, and people with a deficient immune system. Besides bacterial contamination of toothbrushes in general, the occurrence of antibiotic resistance (ABR) might be of interest as well, since antibiotic resistance genes (ARGs) as well as class 1 integrons as an indicator of ABR have been detected in the domestic environment [19,20,21] as well as in the oral microbiome [22,23,24].

A systematic literature review of Frazelle and Munro on microbial contaminations of toothbrushes revealed only few experimental studies [3], and other publications mostly evaluated toothbrush hygiene of children [18,25,26,27]. However, most of the studies focus on the presence of species causing oral diseases like *Streptococcus mutans* and do not differentiate between bristle materials. Therefore, this study aimed to analyse the microbiological contamination of toothbrushes quantitatively and qualitatively with regards to the period of use and user age. We determined the bacterial load, abundance of ARGs, and the composition of the microbiome on toothbrushes using both a cultural and a metagenomic approach. Based on the results of the microbiome analysis, we conducted an in-vitro test simulating the use of toothbrushes over a one-week period with *Bacillus subtilis* (*B. subtilis*), *Klebsiella pneumoniae* (*K. pneumoniae*), *Micrococcus luteus* (*M. luteus*), *Pseudomonas aeruginosa* (*P. aeruginosa*), *Streptococcus anginosus* (*S. anginosus*), and *Streptococcus mutans* (*S. mutans*) to evaluate the effect of long-term use and different bristle materials on microbial survival.

## 2. Material and Methods

### 2.1. Sampling

All samples were collected in May 2019. For all samples, the owners of the households gave permission to conduct the study on these sites. According to German law, no formal ethical review is required for the investigation of articles of daily use. Toothbrushes of 25 persons from different households in North Rhine-Westphalia (Germany) were probed. All toothbrushes were sampled within 24 h after the last use. In addition, a questionnaire was given to consumers to collect completely anonymous information on the general conditions such as age of users and duration of use. Out of the 25 toothbrushes, 11 had been used for four to 12 weeks, six for less than two weeks, six for two to four weeks, and two for more than 12 weeks. Furthermore, the age ranged from 10 to >60 years. All participants stated to be free of any oral or systemic diseases and no further characteristics such as sex or toothbrush type were collected.

### 2.2. Sample Preparation

The toothbrush heads were cut off and transferred to a 50 mL tube containing 20 mL of sterile 0.9% sodium chloride, followed by vigorously vortexing for one minute and incubation for 20 min on a tilt/roller mixer. After removal of the toothbrush heads, samples were centrifuged at 4816× *g* for 20 min and the pellet was re-suspended with 3 mL of 0.9% sodium chloride. Samples were serially diluted and 100 µL of each dilution were plated on tryptic soy agar (TSA; Merck, Darmstadt, Germany), malt extract agar (MEA; Oxoid, Hampshire, UK), Columbia blood agar (Oxoid, Hampshire, UK), and MacConkey agar (Carl Roth, Karlsruhe, Germany) followed by incubation for 48 h at 30 °C (MEA), 37 °C (TSA and MacConkey), and 37 °C under anaerobic conditions (Columbia blood agar) and both aerobic and anaerobic conditions (TSA) in order to estimate the total viable count (TVC) in cfu (colony forming units per toothbrush).

(1) $$C_{wei}=\;\frac{\sum^{\;}C}{\left(n_{1}*1\right)+\left(n_{2}*0.1\right)}*d*10$$

*C_wei_* = weighted arithmetic average. ∑*C* = sum of viable cell count of all agar plates, used for calculation. *n*_1_ = count of agar plates with the lowest evaluable dilution. *n*_2_ = count of agar plates of the next higher dilution stage. *d* = dilution factor of the lowest evaluable dilution stage.

### 2.3. DNA Extraction

For purification of total DNA, the Fast DNA Spin Kit for Soil (MP Bio, Santa Ana, CA, USA) was used according to the manufacturer’s instructions with the following adjustments as performed before [19,28].

Instead of 500 mg solid material, 250 µL of suspended sample were applied to the lysing matrix tube. Samples were homogenized twice in the FastPrep-24™ instrument for 60 s at 6.0 m/s. All samples were washed twice using the SEWS-M solution of the kit. After DNA-extraction, real-time quantitative PCR was performed for the detection of resistance genes and samples were sent to Eurofins Genomics for microbiome profiling using NGS (Next generation sequencing) [19].

### 2.4. Detection of Bla, Mcr, and IntI1 Genes

To detect *bla* and *mcr* genes, real-time quantitative PCR (qPCR) was performed as described before [20]. All sequences of the used oligonucleotides (custom synthesized by Biolegio, Nijmegen, Netherlands) are shown in Table 1. The genes *bla*_ACT_ and *bla*_MIR_ will be referred to as *bla*_ACT/MIR_, since the oligonucleotides targeted both. The prepared DNA was amplified using the HotStarTaq^®^ Master Mix Kit (Qiagen, Hilden, Germany) and a mix of oligonucleotides and DNA probes labelled with different dyes. The PCR mix contained 10 µL of a 2× HotStarTaq^®^ Mastermix, 7 µL of RNase-free water, 1 µL of oligonucleotide mix (oligonucleotide: 4 µM; probe: 2 µM and MgCl_2_: 50 µM), and 2 µL of the DNA template comprising a final volume of 20 µL. The PCR was performed on a LightCycler 480 (Roche Life Sciences, Mannheim, Germany) using the following conditions: 95 °C for 15 min, 45 cycles of denaturation (95 °C; 10 s), annealing (60 °C; 20 s), elongation (72 °C; 10 s), and a final cycle at 30 °C for 30 s. A sample was considered positive if it reached the threshold before cycle 40 or if the copies/µL determined by standard curve were greater than five copies/µL. For determination of 16S ribosomal DNA (16S rDNA) and *intI1* genes, qPCR was performed according to Lucassen et al. (2019) [19]; primers are shown in Table 2.

### 2.5. Microbiome Profiling

Since the study aimed to analyse the microbial community on toothbrushes, the INVIEW Microbiome Profiling 3.0 from Eurofins Genomics (Eurofins Genomics Germany GmbH, Ebersberg, Germany) was chosen for a NGS-based taxonomic analysis of the samples. The analysis targeted the 16S regions V1–V3 of bacterial DNA with an output of 10 million read pairs per sample and bioinformatics was performed by Eurofins Genomics as well. Since no or rather weak amplicons were generated for the samples TB6 and TB7, these samples were excluded from sequencing.

### 2.6. In-Vitro Test of Different Toothbrushes

To examine the attachment and survival of bacteria on toothbrushes and the effect of different bristle materials on microbial growth, toothbrushes were artificially contaminated twice a day with six different test strains for a one-week period. A total of five different toothbrushes with different bristle types were tested comprising the materials nylon, pig bristles, bamboo viscose, bio-nylon, and charcoal with nylon. The toothbrushes were immersed in 15 mL bacterial suspension (approx. 10^8^ cfu·mL^−1^) in a 50 mL tube for two minutes each morning and evening, in order to simulate the time and frequency of routine tooth brushing. *Bacillus subtilis* (DSM 1088), *Klebsiella pneumoniae* (DSM 26371), *Micrococcus luteus* (DSM 1790), *Pseudomonas aeruginosa* (DSM 939), *Streptococcus anginosus* (DSM 20563), and *Streptococcus mutans* (DSM 20523) were selected as test bacteria because they were identified in metagenome analysis as frequently occurring microorganisms on toothbrushes in private households. After incubation in the bacterial suspension, the toothbrushes were stored upright at room temperature between tests (approx. 8 h to simulate realistic conditions).

After the test period, the toothbrushes were treated as described above and plated on TSA, MacConkey, and Columbia blood agar. The plates were incubated for 48 h at 37 °C (TSA plates) and 37 °C, microaerophilic (Columbia blood agar). Each test was performed in triplicate for each bacterial strain.

### 2.7. Statistical Analysis

Statistics were performed using GraphPad Prism (GraphPad Software, Inc.). Data were expressed as means (±standard error). Data of microbial counts on toothbrushes were normally distributed (as confirmed by Shapiro-Wilk test). Thus, statistically significant differences were assessed using multiple t-test or two-way analysis of variance (ANOVA) and post-hoc Tukey’s multiple comparison test was performed to identify significant differences between the bacterial growth on the different culture media as well as the different bristle materials (*p ≤* 0.05). Since relative abundance of beta-lactamase (*bla*) genes was not normally distributed (Shapiro-Wilk test), non-parametric Kruskal-Wallis test (*p ≤* 0.05) was performed to identify significant differences between the targeted *bla* genes. Shannon-Index was calculated to determine alpha diversity and weighted UniFrac distance analysis of the microbiomes was used to compare beta diversity among samples.

## 3. Results

### 3.1. Taxonomic Distribution

**Figure 1:**
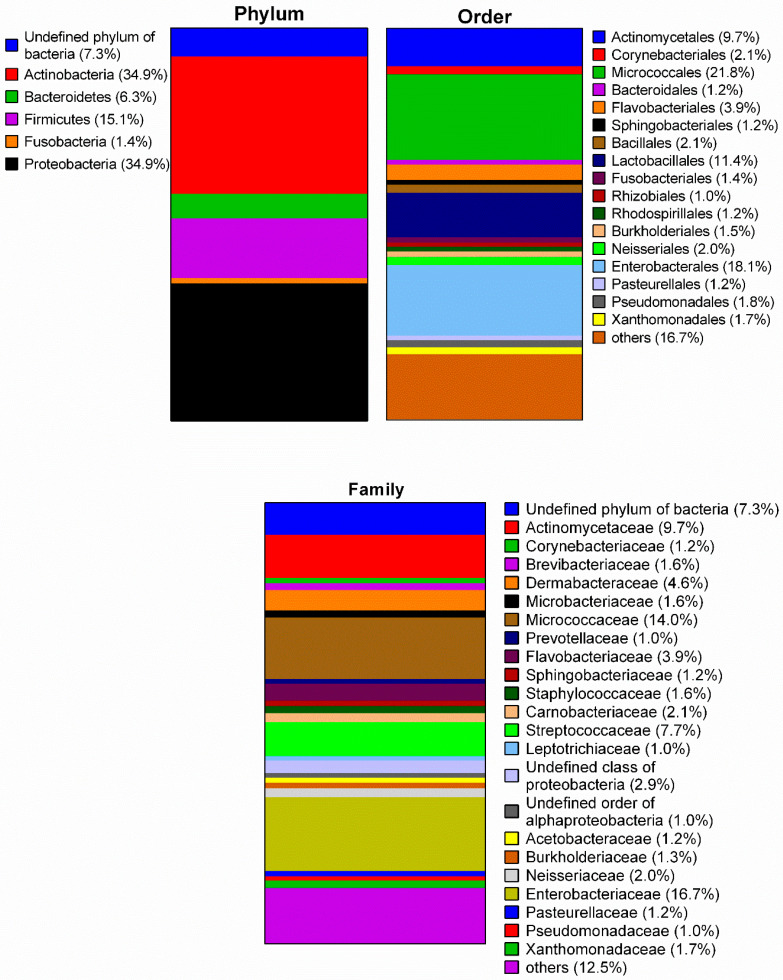
Taxonomic composition of the toothbrush microbiome based on next generation sequencing of 23 toothbrush samples. To ameliorate presentation, only results ≥1% are given.

A total of 23 toothbrush microbiomes were analysed using NGS, revealing 1,581,052 sequences assigned to 2,341 OTUs (operational taxonomic units). Figure 1 indicates the average microbial composition of the toothbrush samples at phylum-, order- and family-level. The analysis revealed nine phyla, 22 classes, 43 orders, 74 families, and 130 genera. The majority of identified bacteria belonged to the phyla *Actinobacteria* (34.9%) and *Proteobacteria* (34.9%) while the phyla *Deinococcus-Thermus*, *Streptophyta*, and *Cyanobacteria* were poorly represented (<1.0%). Thus, the classes *Actinobacteria* and *Gammaproteobacteria* dominated with 34.6% and 23.1%, respectively (see Table S1). The order *Micrococcales* occurred most (21.8%) followed by *Enterobacterales* (18.1%), *Lactobacillales* (11.4%), and *Actinomycetales* (9.7%). *Micrococcaceae* (14.0%) were the most abundant family among *Actinobacteria*, *Streptococcaceae* (7.7%) among *Bacilli*, and *Enterobacteriaceae* (16.7%) among *Gammaproteobacteria*.

**Figure 2:**
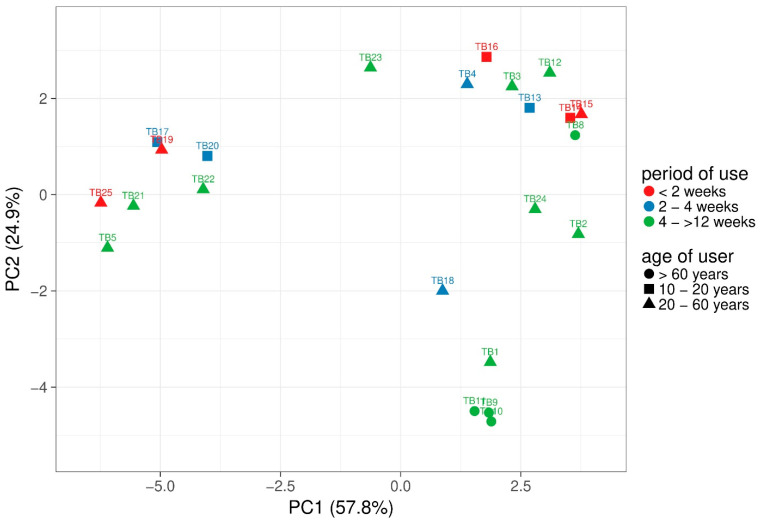
Principal component analysis (PCA) of toothbrush samples (*n* = 23) based on weighted UniFrac distance analysis. Unit variance scaling was applied to rows and NIPALS (Nonlinear Iterative Partial Least Squares) PCA was used to calculate principal components. X- and Y-axis show principal component 1 and principal component 2 that explain 57.8% and 24.9% of the total variance, respectively. Period of use: <2 weeks (*n* = 6), 2–4 weeks (*n* = 6), 4–>12 weeks (*n* = 11)) and user age: (10–20 years (*n* = 5), 20–60 years (*n* = 14), >60 years (*n* = 4)).

Alpha-diversity was calculated based on observed OTUs. Between 83 and 256 different species were observed per sample and Shannon index of the samples ranged from 3.45 to 7.06. However, non-parametric Kruskal-Wallis test revealed no significant differences of alpha diversity based on user age or period of use. To compare the different samples, principal component analysis (PCA) with weighted UniFrac distance analysis of the microbiomes was performed using ClustVis (Figure 2) [31]. Samples are coloured based on period of use and the different age is presented as shapes. The majority of the samples with short distances revealed strong variations of age and period of use. However, TB17 and TB19 clustered closely together and revealed a rather short period of use with two to four weeks and shorter than two weeks, respectively. Most of the samples TB3, TB4, TB8, TB12, TB13, TB14, TB15, and TB16 were used for a short period (<2 to 4 weeks) and although TB15, TB14, and TB8 were clustered closely together, only TB15 and TB14 were used for the same period and the age differed between all three samples. In contrast, TB24 and TB2 as well as TB9, TB10, and TB11 revealed the same period of use and age group.

Considering the order-level, *Micrococcales* occurred in all samples (TB17, TB21 and TB22 < 1.0%) and dominated on four toothbrushes (55–90%) while *Enterobacterales* were detected on 10 toothbrushes varying between 0.7 and 81%. *Rothia* and *Kocuria* were the most common genera of the *Micrococcaceae*, mainly represented by the species *R. dentocariosa* (15 samples) whereas *Kocuria* could not be identified to the species level in most samples. Although the *Enterobacterales* were dominantly *Enterobacteriaceae*, most could not be identified on species level but inter alia *Klebsiella* and *Citrobacter* occurred as genera. Besides, the orders *Actinomycetales* (0.3–42.0%) and *Lactobacillales* (0.1–37.1%) were detected in 22 and 23 samples, respectively. The genus *Actinomyces* (9.0%) was detected in all samples except TB10 with the dominating species *Actinomyces oral taxon 448* (13 samples), *A. massiliensis* (13 samples), and *A. oris* (12 samples). For the *Lactobacillales*, the genus *Streptococcus* (7.7%) occurred in all analysed samples and amongst 44 different *Streptococcus* species in the samples, *S. sanguinis* (18 samples), *S. mitis* (15 samples), *S. cristatus* (12 samples), and *S. oralis* (11 samples) occurred most often. Furthermore, *Haemophilus parainfluenzae* and the *Streptococcus* like bacterium *Granulicatella adiacens* were identified in 17, *Gemella haemolysins* in 14, *Lautropia mirabilis* in 13, and *Fusobacterium nucleatum* as well as *Kingella oralis* in 12 toothbrush samples.

**Figure 3:**
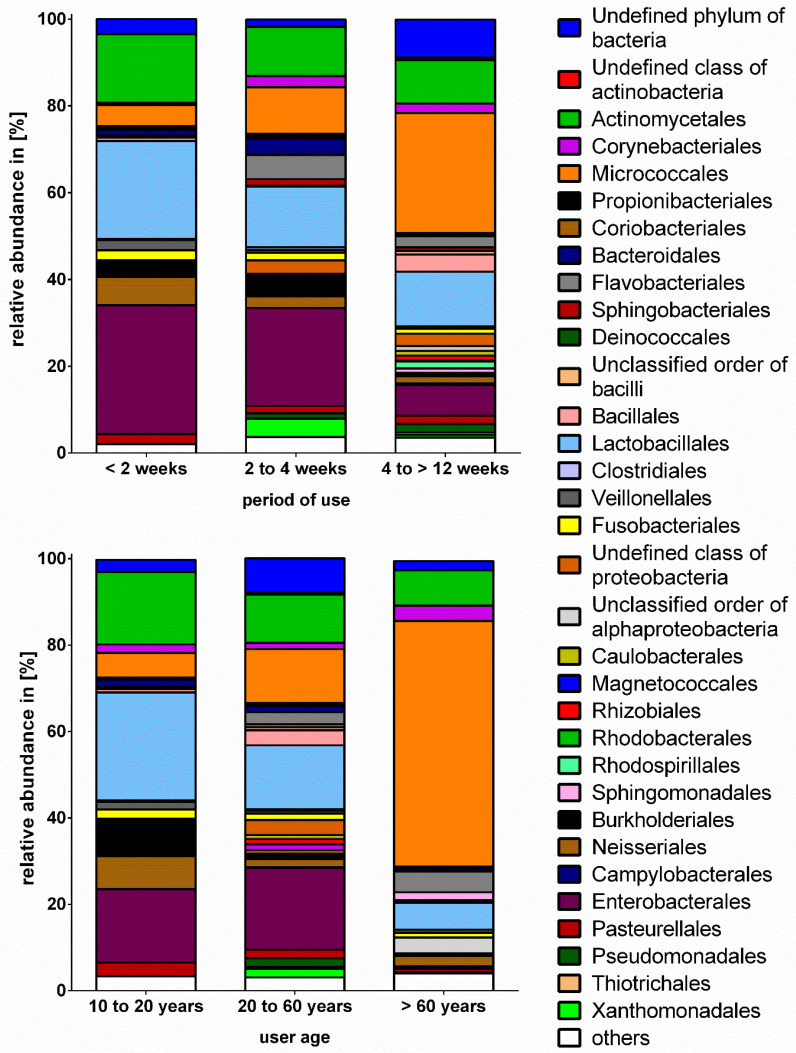
Taxonomic composition on order-level for all analysed toothbrushes grouped by period of use (<2 weeks (*n* = 6), 2–4 weeks (*n* = 6), 4–>12 weeks (*n* = 11)) and user age (10–20 years (*n* = 5), 20–60 years (*n* = 14), >60 years (*n* = 4)) based on NGS. For samples TB6 and TB7, the generation of amplicons failed and therefore these samples were not sequenced. Due to better presentation, only results ≥1% are given.

Due to the low number of toothbrushes used for more than 12 weeks (*n* = 2), samples are presented combined as the group four to >12 weeks. The comparison of the taxonomic composition on order level revealed significant differences between samples grouped by period of use (Figure 3). Although beta-diversity did not differ significantly between samples grouped based on period of use, non-parametric Mann-Whitney test (*p* ≤ 0.05) revealed significant differences based on order level between the usage of less than two weeks and four to >12 weeks. The percentage of *Micrococcales* was significantly higher in the group four to >12 weeks compared to >2 weeks, while the percentage of *Enterobacterales* was significantly lower (Table 1). Moreover, the amount of *Lactobacillales*, *Actinomycetales*, *Enterobacterales*, and *Neisserales* slightly decreased from shortest to longest period of use. Interestingly, the *Pseudomonadales* and *Sphingomonadales* were not detected in toothbrush samples used for less than two weeks whereas toothbrushes used for a period between four and >12 weeks revealed the highest, but still very low percentages of these bacterial orders. When comparing the microbial composition based on user age, the group of >60 years differed significantly from group 20 to 60 years and revealed a significantly higher percentage of *Micrococcales* while no *Enterobacterales* were detected. The *Lactobacillales* dominated in the group of the youngest participants and the *Enterobacterales* were slightly lower compared to the group 20 to 60 years. The prevalence of *Micrococcales* seemed to increase with increasing age and *Pseudomonadales* only occurred in the groups 20 to 60 and >60 years. However, *Lactobacillales* and *Actinomycetales* decreased from the group 10 to 20 years to the group of >60 years (Table 3).

### 3.2. Microbial Contamination of Toothbrushes

A total number of 25 toothbrushes with varying period of use and user age (Table 4) was analysed to determine the microbial contamination using culture-based approaches. The means of total viable counts (TVC) under aerobic and anaerobic conditions as well as the amount of *Streptococcus* spp., gram-negatives and fungi are shown in Table 5. High bacterial counts (1.42 × 10^6^ cfu·mL^−1^ to 1.19 × 10^7^ cfu·mL^−1^) were found on all tested media.

**Figure 4:**
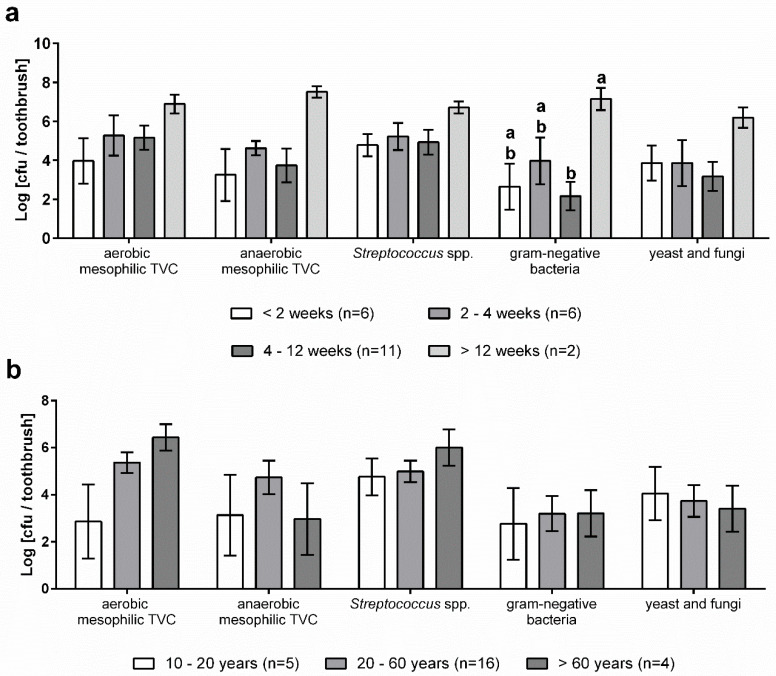
Microbial count of toothbrushes grouped by period of use (**a**) and user age (**b**) on different culture media. Columns show the mean and standard error of mean (*n* = 25). Different letters indicate significant differences at *p* ≤ 0.05 (multiple *t*-test) between groups. Where no letters are shown, no significant differences were detected.

Figure 4 shows the microbiological contamination of toothbrushes in relation to the age of users and the period of use. Although the aerobic mesophilic TVC of toothbrushes of older users was much higher compared to younger users, the means did not differ significantly (*p* = 0.9). In contrast, the other media tested revealed only minor differences between the age groups (Figure 4b). The anaerobic mesophilic TVC was highest in samples of the age group between 20 and 60 years and the microbial count of *Streptococcus* spp. was higher in samples belonging to the group of >60 years.

In addition, the microbial counts on toothbrushes were evaluated grouped by the period of use (Figure 4a). The highest bacterial loads were determined for a period of use of more than 12 weeks on all media tested (3.25 × 10^5^ cfu·toothbrush^−1^ to 7.50 × 10^6^ cfu·toothbrush^−1^) and the count of gram-negative bacteria was significantly higher compared to the other periods. However, no trends for the other use periods were identified.

### 3.3. Occurrence of Antibiotic Resistance Genes

Besides NGS, qPCR for the screening of beta-lactamase (*bla*), mobile colistin resistance (*mcr*), and class 1 integron integrase (*intI1*) genes was performed. As a result of our investigation, in 20 of the analysed samples, *bla* genes were detected; while no *mcr* genes occurred and all samples, except TB6 and TB14, revealed the presence of class 1 integrons. Of the 13 *bla* types chosen for screening, only *bla*_ACT/MIR_, *bla*_CMY-2_, *bla*_GES_, *bla*_KPC_, *bla*_OXA-48_, *bla*_OXA-23_ and *bla*_OXA-58_ occurred in the toothbrush samples (Table 6). *Bla*_OXA-58_ was most frequently identified, followed by *bla*_OXA-23_ and *bla*_GES_.

**Figure 5:**
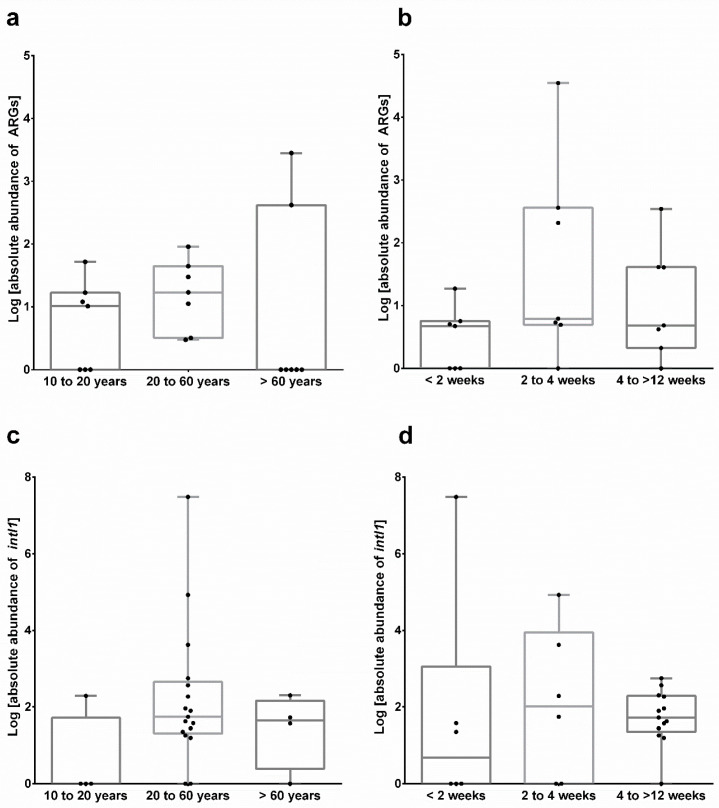
Absolute abundance of total ARGs (**a**,**b**) and *intI1* (**c**,**d**) of toothbrush samples grouped by age (10–20 years (*n* = 5), 20–60 years (*n* = 16), >60 years (*n* = 4)) and period of use (<2 weeks (*n* = 6), 2–4 weeks (*n* = 6), 4–>12 weeks (*n* = 13)). Analyses were performed in duplicate and non-parametric Kruskal-Wallis test (*p ≤* 0.05) revealed no significant differences between sample groups.

The absolute abundance of both total ARGs and *intI1* varied strongly across samples, thus no significant differences were detected (Figure 5). However, in all samples of the group, 20 to 60 years ARGs were detected and *intI1* revealed the highest abundance as well. Despite a shorter period of use, toothbrushes used between two and four weeks revealed the highest values of both ARGs and *intI1*.

### 3.4. In-Vitro Study of Different Types of Toothbrush Bristles

**Figure 6:**
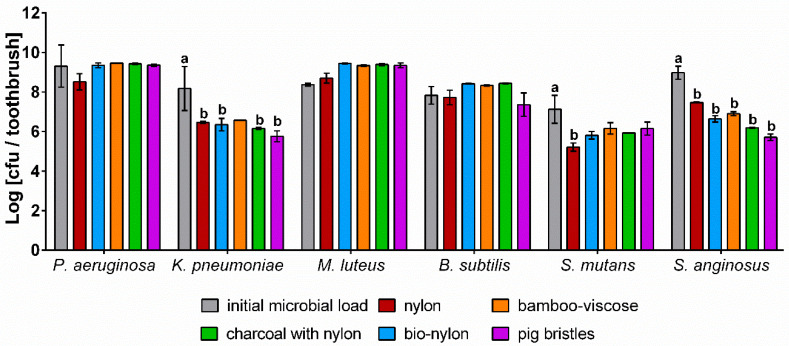
Logarithmic microbial counts of different bristle types after one week of artificial contamination. The following strains were used: *Pseudomonas aeruginosa* (*P. aeruginosa*), *Klebsiella pneumoniae (K. pneumoniae*), *Micrococcus luteus* (*M. luteus*), *Bacillus subtilis* (*B. subtilis*), *Streptococcus mutans* (*S. mutans*), and *Streptococcus anginosus* (*S. anginosus*) (*n* = 3). Different letters indicate significant differences at *p* ≤ 0.05 between groups. Where no letters are shown, no significant differences were detected.

To examine the survival and retention of bacteria on toothbrushes and the effect of different bristle materials on microbial growth and survival, toothbrushes were artificially contaminated twice a day with six different microorganisms (Figure 6).

All test strains were detected on the toothbrushes after a period of one week with bacterial loads close to the initial count of the bacterial suspensions. None of the bristle types tested revealed a particular antimicrobial effect, although charcoal bristles and bamboo bristles are supposed to have antimicrobial properties. However, no positive influence on bacterial growth was observed as well. While the lowest bacterial counts were determined for either nylon or pig bristles, the other bristle materials showed nearly no differences. Although the bristle type only had a minor effect, the test strains differed in growth. In case of *P. aeruginosa*, *M. luteus*, and *B. subtilis*, the TVC on the tested bristle types slightly increased or revealed the same amount compared to the microbial load of the bacterial suspension. In contrast, lower TVCs of *K. pneumoniae*, *S. mutans*, and *S. anginosus* remained on all bristle types, even revealing significant differences compared to the bacterial suspension. Similar to the NGS results, the in-vitro tests showed an increase of *Micrococcales* on the toothbrushes as well, while *Streptococcus* spp. and *Enterobacteriaceae* revealed lower bacterial loads.

## 4. Discussion

The current study aimed to analyse the microbial contamination of toothbrushes and to determine possible factors impacting microbial contaminations. The mean TVC (aerobic and anaerobic) as well as the mean counts of *Enterobacteriaceae*, *Streptococci*, and fungi within the present investigation ranged from 1.42 × 10^6^ cfu·mL^−1^ to 1.19 × 10^7^ cfu·mL^−1^ and our results are generally consistent with other studies [13,18,32]. Using NGS, the current investigation suggests that *Actinobacteria* and *Proteobacteria* dominate the toothbrush microbiome, mainly represented by *Micrococcales*, *Enterobacterales, Lactobacillales*, and *Actinomycetales*. While dental pathogens only occurred in a low fraction, toothbrushes were commonly contaminated with *Enterobacteriaceae*.

### 4.1. Bacterial Diversity of Toothbrushes

As far as we know, this study is the first comprehensive, culture-independent analysis of the toothbrush microbiome. Previous studies focused on microbiological characterization regarding bacterial load [13,27], biofilm formation [18], or dental pathogens [33,34]. Although dental pathogens such as *Streptococcus mutans*, *Fuseobacterium nucleatum*, *Prevotella* spp. or *Eikenella corrodens* [35] were found in our study as well, these species only occurred in less than half of the samples and in small fractions (0.1–0.9% of all samples) of the total microbiome. The relative abundance of the taxa varied strongly across the toothbrush samples; only taxa like *Micrococcales*, *Lactobacillales* and *Actinomycetales* were detected in nearly all samples. The majority of the identified bacterial species are common residents of the oral cavity, like *Micrococcaceae* (e.g., *Rothia dentocariosa*), *Streptococcaceae* (e.g., *S. mitis*), or *Actinomycetaceae* (e.g., *Actinomyces* spp.) and thus could be expected on toothbrushes [36]. The variation might be due to differences between the underlying households in, e.g., storage conditions of toothbrushes, since studies showed increased contaminations on toothbrushes stored in closed containers or humid environments [1,37]. Apart from influencing the mere number of microbial cells that are present on toothbrushes, numerous factors, like the period of use of the toothbrush, age of the user, diet, cleaning habits, etc., might also affect the composition of the microbial community. This is supported by the significant differences of the microbial composition of samples grouped by period of use or user age, although alpha- and beta-diversity did not differ significantly. Toothbrushes used between four and longer than 12 weeks revealed the presence of species involved in biofilm formation in domestic environments [38,39] such as *Pseudomonadales* and *Sphingobacteriales*, which might be built up on toothbrushes in a similar manner. Furthermore, the prevalence of *Micrococcales* increased with longer period of use. In contrast, taxa of the oral microbiome like *Lactobacillales* (e.g., *Streptococcus* spp.), *Neisseriales* (e.g., *Neisseria mucosa*), and *Actinomycetales* [35] occurred less frequently with increasing use. These shifts in the microbial composition might be connected to dominant growth of specific taxa over time, which are better adapted to the toothbrush environment. For example, *Micrococcales* like *Kocuria* spp. and *Rothia* spp. grow aerobically in both the environment and the oral cavity [40,41] while *Streptococci* or *Actinomyces* spp. usually prefer an anaerobic environment, which is not provided by a toothbrush.

The significant differences between different age groups might at least be partially related to changes of the oral microbiota with increasing age [42,43]. Xu et al. (2015) [44] showed that the abundance of *Firmicutes* was higher at younger age, which might explain that *Lactobacillales* dominated on toothbrushes of younger age groups. Furthermore, their study showed that the relative abundance of *Actinobacteria* increased steadily from the age 15 to 76 and this trend was observed for *Micrococcales* in the toothbrush samples as well. Clustering of samples based on weighted Unifrac revealed at least partially small distances between samples of the same user age/period of use, indicating that these microbiomes had many similarities. However, other factors might be responsible for the differences as well since samples without the same user age/period of use clustered closely together as well and thus further investigations are needed.

The results indicate that a recontamination of the oral cavity during the use of contaminated toothbrushes seems likely and might result in possible health problems [2,3,11]. Besides species of the oral microbiome, opportunistic environmental bacteria of the *Pseudomonadaceae* (1.0%) and especially the *Enterobacteriaceae* (16.7%) occurred in the toothbrush samples, suggesting a putative contamination from the environment or aerosols from toilet flushing in bathrooms [3,5]. In most of the toothbrush samples harbouring high percentages of the *Enterobacteriaceae* species like *Klebsiella* spp., *Citrobacter* spp. or *Enterobacter* spp., were detected. Contreras et al. (2010) [5] determined high contaminations of toothbrushes with species of *Enterobacteriaceae* and *Pseudomonadaceae*, while the fractions of dental pathogens were low, which is consistent with our study. Thus, the toothbrush might serve as a reservoir of these opportunistic pathogens and a recontamination of the oral cavity might result in infections. This is especially of great concern regarding the increasing number of elderly or ill people being nursed at home.

### 4.2. Microbial Contamination of Toothbrushes

Our results showed that both the mean aerobic and anaerobic TVC was approximately 10^7^ cfu/toothbrush. The values for *Streptococcus* spp. and gram-negative bacteria were ten-fold lower, suggesting that other groups account for a majority of bacterial cells present on the toothbrush. The *Streptococcus* spp. described in this section include all microbiologically identified streptococci that have grown on the selective agar. In comparison to NGS, allowing an identification on species level such as *Streptococcus mutans* among the dental pathogens [33,34], non-pathogenic Streptococci might have also been isolated in the microbiological analysis. In general, the current investigation revealed slightly higher counts than those found in previous studies. While both Sammons et al. (2004) and Malta et al. (2019) found that the aerobic TVC on toothbrushes was approx. 10^6^ cfu [18], Taji and Rogers determined an aerobic mesophilic TVC between 10^4^ cfu/toothbrush and 10^6^ cfu/toothbrush [13,32]. Of the toothbrushes analysed, 56% were contaminated with gram-negative bacteria and the NGS showed that approx. 16.7% of gram-negative bacteria were *Enterobacteriaceae*. Since gram-negative bacteria are not typical colonizers of the oral cavity [36], their occurrence indicates that the source for toothbrush contamination is not exclusively the oral microbiome. Although not determined in this study, the humidity and aerosols in the bathroom environment most likely facilitated these contaminations [3,5].

In addition to the available information from the literature [42,43,44], the present study suggests that the bacterial colonization of toothbrushes seems to be dependent on user age. Although there was no significant difference, the toothbrushes used by people of over 60 years exhibited the highest aerobic mesophilic TVC and the highest numbers of *Streptococcus* spp. In contrast, the anaerobic TVC was highest in the age group between 20 and 60 years.

A further aspect of this study was to investigate the bacterial contamination over the period of use of the toothbrushes. Here, the aerobic TVC tends to increase slightly from zero to two weeks and almost stagnates between the third and twelfth week. From the twelfth week, the bacterial load again increased strongly (*p* = 0.9) to a maximum of approx. 10^7^ cfu/toothbrush. A similar effect was observed for other microbial groups, revealing a major increase in microbial contamination for toothbrushes that have been used for more than 12 weeks. Because the exact period of use has not been determined, it cannot be excluded that the increase is following a linear function. Nevertheless, our results show clearly that toothbrushes are able to accumulate a high number of microorganisms over time and thus should be changed regularly (i.e., within less than three months). A German consumer survey found that every third person changes the toothbrush every two to three months [45]. However, about 16% of the people in Germany change their toothbrush less often. A similar study by Ziebolz et al. (2006) showed that almost 25% of Germans use their toothbrush for longer than 12 weeks [46]. Thus, since pathogenic microorganisms are also present on toothbrushes, adverse microbial effects might be more likely if toothbrushes are not exchanged regularly [34]. Studies showed that the bristle material, toothbrush design, or toothpaste can impact the microbiological contamination of toothbrushes [1,10,47,48]. On the other hand, it has been shown that toothbrushes with antibacterial coating did not inhibit bacterial growth [10,48]. However, no data regarding the nature of the bristles, density, or hardness were collected in our study and thus an effect of these parameters cannot be ruled out and should be taken into account in further investigations.

### 4.3. Occurrence of Bla and IntI1 Genes in Toothbrush Samples

Few previous studies analysed the frequency of ABR bacteria [21] and the occurrence of *bla* genes [20] in the domestic environment and the oral microbiome [22,23,24]. Lucassen et al. (2019) [19] determined the correlation of the presence of multi-resistance bacteria and *intI1*. This study also showed a strong variation of all *bla* genes across toothbrush samples, with the highest abundance of *intI1* and *bla*_OXA-58_ across the samples. In contrast to Rehberg et al. (2017) [20], who found a high prevalence of ampC-β-lactamase genes in different household samples, only *bla*_CMY-2_ and *bla*_ACT/MIR_ occurred in five and four samples in the present study, respectively. This might be due to different compositions of the microbial community of the samples. While ampC-β-lactamases occur frequently in biofilms of aquatic environments [49] and are especially produced by *Enterobacteriaceae* [50], these species were detected less frequently (16.7%). Nevertheless, *bla*_OXA-58_ was often detected in washing machines and dishwashers of this previous study as well and these genes dominated in the analysed toothbrush samples of the present study. The higher abundance of ARGs that we have found in the current study in samples of users between 20 and 60 years might be caused by a higher diversity of the microbiome. Furthermore, samples of this age group comprised of a higher percentage of *Enterobacteriaceae* and *Pseudomonadaceae*, which are known to commonly harbour *bla* and *intI1* genes [51]. The lower abundance in the youngest age group might be connected to antibiotic exposure, since the times of antibiotic intake are usually lower at younger age and ABR seems to increase with increasing age [52]. Toothbrushes used between two and four weeks revealed the highest abundances of ARGs and *intI1*, although the number of samples was higher in the group four to >12 weeks. Since the microbiomes of this group exhibited a higher amount of gram-negative bacteria compared to the longest period of use, this might explain the higher amount of ARGs. However, due to the high prevalence of gram-positive species in the toothbrush samples and since the targeted *bla* genes mainly occur in gram-negative bacteria [50,53], a lower frequency of the ARGs compared to other environments seems reasonable.

### 4.4. In-Vitro Study of Different Bristle Materials

The results of the artificial contamination of toothbrushes showed that the bristle type had no significant influence on the bacterial growth and survival, although bamboo and charcoal are supposed to have antimicrobial properties. While Thamke et al. (2018) [4] determined an antimicrobial impact of charcoal toothbrushes on anaerobic bacteria, our results showed no considerable differences compared to the other bristle types. Although toothbrush made from animal bristles ought to be prone to contaminations, no significant differences compared to other bristle types were observed. Thus, it can be assumed that the use of natural bristles does not pose a microbiological risk. Especially environmental bacteria like *P. aeruginosa* and *M. luteus* remained on the different bristle types and a formation of colonies on the bristles was already observed after one week (data not shown). An increasing prevalence of *Micrococcales* with longer periods of use was observed in the microbiome analysis as well. Since pure cultures were used in the in-vitro study, the results indicate that rather the material of the toothbrush or growth conditions of the test strain than competition of growth are responsible for the observed trend. The colony formation indicate rapid biofilm formation on the toothbrushes, which has already been confirmed by Sammons et al., determining a rapid biofilm formation of *P. aeruginosa* on toothbrushes after a few days [18]. In contrast, *S. mutans* and *S. anginosus* revealed lower TVCs on the tested bristles, indicating a worse survival/attachment compared to the other test strains. This is confirmed by the results of NGS showing decreasing amounts of *Lactobacillales* with increasing period of use. Since *Streptococcus* spp. grow preferably under anaerobic conditions, an impaired growth seems reasonable. However, the results revealed that *S. mutans* and *S. anginosus* still remained on the toothbrushes and thus a recontamination of the oral cavity might be possible.

The survival of *S. mutans* on toothbrushes has already been investigated in various studies [3,14,16,34], showing that *S. mutans* is able to grow on bristles and even to form biofilms. Ferreira et al. (2012) [17] and Contreras et al. (2010) [5] determined very high contaminations of toothbrushes with gram-negative bacteria and our results confirmed that both *Enterobacteriaceae* and *Pseudomonadaceae* survive and even grow on toothbrushes. Hence, the storage of toothbrushes separated from the bathroom environment might prevent putative contaminations with gram-negative bacteria.

## 5. Conclusions

The results obtained in this study substantiate that toothbrushes represent a potential reservoir of opportunistic environmental bacteria and, although in smaller fractions, dental pathogens. Compared to other environments, we found a lower frequency of *bla* and *intI1* genes, which indicates a lower resistance potential. We observed that all tested toothbrushes were highly contaminated and bacterial counts were highest after a use of more than 12 weeks. Especially the *Micrococcales* dominated in the samples and increased over time. This trend was not only observed via NGS but also when performing in-vitro tests, indicating that species of the *Micrococcales* occur frequently on toothbrushes and grow predominantly with and without the presence of other bacterial species. In contrast, the frequency of *Lactobacillales* (e.g., *Streptococcus* spp.) slightly decreased with increasing period of use, which was confirmed by the in-vitro tests with *S. mutans* and *S. anginosus*. Moreover, our data show that toothbrushes partially had similar beta-diversities if used for the same period or of the same age group, indicating that both user age and period of use might influence the development of the microbial community. However, to identify the responsible factors influencing the composition of the toothbrush microbiome, further investigations are mandatory. Based on the present study, toothbrushes should be replaced after three months at the latest, and in the best case after one or two months.

## Figures and Tables

**Table 1:** Primers used for the detection of genes encoding beta-lactamases (*bla*) and mobile colistin resistance (*mcr*).

Primer	5′–3′
KPC-1	GCCGTGCAATACAGTGATAAC
KPC-2	GAACGTGGTATCGCCGATAG
KPC-probe	TexRed-CCGCCGCCAATTTGTTGCTGAAGG-BHQ2
OXA48-1	GTTGGAATGCTCACTTTACTGAA
OXA48-2	TTCGCCCGTTTAAGATTATTGG
OXA48-probe	FAM-ATTCTCATTCCAGAGCACAACTACGCC-BHQ1
GES-1	CTCTGTGAGTCGGCTAGACC
GES-2	CGATCAGCCACCTCTCAATG
GES-probe	HEX-ACACCTGGCGACCTCAGAGATACAACT-BHQ1
VIM-1	GTTTGGTYGCATATCGCAAC
VIM-2	CTTYTCAATCTCCGCGAGAAG
VIM-probe1	FAM-AGCAACTCATCRCCATCACGGACAATG-BHQ1
VIM-probe2	FAM-AACTCGGTGACACGGTGTACTCGTCT-BHQ1
NDM-1	CGACTTATGCCAATGCGTTG
NDM-2	CGGGGTAAAATACCTTGAGC
NDM-probe	HEX-AGCCTGACTTTCGCCGCCAATG-BHQ1
OXA23-1	CCTGATCGGATTGGAGAACC
OXA23-2	GTTCCTGATAGACTGGGACTG
OXA23-probe	FAM-TGGCTTCTCCTAGTGTCATGTCTT-BHQ1
OXA58-1	GTTGGTATGTGGGTTTTGTTG
OXA58-2	CGTAGAGCAATATCATCACCAG
OXA58-probe	Cy5-TGCCACCACTTGCCCATCTGCC-BBQ^®^-650
CTXM-1-1	AATCTGACGCTGGGTAAAGC
CTXM-1-2	GATATCGTTGGTGGTGCCATA
CTXM-1-Probe	FAM-CCTGAATGCTCGCTGCACCGG-BHQ1
CTXM-9-1	CCGATCTGGTTAACTACAATCC
CTXM-9-2	GCTGGGCAATCAATTTGTTC
CTXM-9-probe	TexRed-CAACGGCACAATGACGCTGGC-BHQ2
CMY2-1	GATGCAGGAGCAGGCTATTC
CMY2-2	AACACGCCGTTAAACGTCTTAC
CMY2-probe	FAM-CCAATAACCACCCAGTCACGCAG-BHQ1
FOX-1	GTTCGAGATTGGCTCGGTCA
FOX-2	CACTGTAGGTGGCAAGCTCG
FOX-probe	HEX-TGGCTCACCTTGTCATCCAGC-BHQ1
ACT_MIR-1	ACTGGCAGCCGCAGTGGAAG
ACT_MIR-2	ACGTTAATCCASGTATGGTCCAGC
ACT_MIR-probe	FAM-AGACCCGCGTCGTYATGGCCTG-BHQ1
DHA-1	GGCGATATGCGTCTGTATGC
DHA-2	GTCAGCAACTGCTCATACGG
DHA-probe	TexRed-CCTGTTTGGTGCTCTGACCGC-BHQ2
mcr-1-1	ATGGCACGGTCTATGATACG
mcr-1-2	CACACCCAAACCAATGATACG
mcr-1-probe	FAM-ACCGACCAAGCCGAGACCAAGGA-BHQ1
mcr-2-1	GCCAACAGACACCATCTATC
mcr-2-2	TAGCCATTGAACTGCACATG
mcr-2-probe	HEX-ACCACCAAGCCGAGCGAGCG-BHQ1

**Table 2:** Primers used for the detection of class 1 integron-integrase gene (*intI1*) and 16S ribosomal DNA (16S rDNA).

Primer	5′–3′
*intI1* F165	CGAACGAGTGGCGGAGGGTG [29]
*intI1* R476	TACCCGAGAGCTTGGCACCCA [29]
16S rDNA F919	GAATTGACGGGGGCCCGCACAAG [30]
16S rDNA R1378	CGGTGTG TACAAGGCCCGGGAACG [30]

**Table 3:** Percentage of bacterial orders based on period of use and age of the users. Significantly higher fractions are marked with a * based on non-parametric Mann-Whitney test (*p* ≤ 0.05).

Bacterial Order	<2 Weeks (*n* = 6)	2 to 4 Weeks (*n* = 6)	4 to >12 Weeks (*n* = 13)	10 to 20 Years (*n* = 5)	20 to 60 Years (*n* = 16)	>60 Years (*n* = 4)
*Micrococcales*	4.9%	10.8%	27.6% *	5.6%	12.5%	56.9%
*Enterobacterales*	29.7% *	22.6%	7.0%	17.0%	19.0%	0.0%
*Lactobacillales*	22.6%	14.0%	12.6%	25.0%	14.8%	6.2%
*Actinomycetales*	15.8%	11.3%	10.1%	16.8%	11.1%	8.2%
*Neisserales*	6.5%	2.7%	1.6%	7.7%	1.8%	2.4%
*Pseudomonadales*	0.0%	1.2%	2.0%	0.0%	2.1%	0.25%
*Sphingomonadales*	0.0%	0.2%	1.0%	0.3%	0.8%	0.4%

**Table 4:** Overview of the period of use of toothbrush (TB) samples, the age of the users, and storage conditions from different households in North Rhine-Westphalia (Germany).

Sample	Period of Use	User Age	Storage	Sample	Period of Use	User Age	Storage
**TB1**	4–12 weeks	20–60	upright	**TB14**	<2 weeks	10–20	upright
**TB2**	4–12 weeks	20–60	upright	**TB15**	<2 weeks	20–60	upright
**TB3**	4–12 weeks	20–60	upright	**TB16**	<2 weeks	10–20	upright
**TB4**	2–4 weeks	20–60	upright	**TB17**	2–4 weeks	10–20	horizontal
**TB5**	4–12 weeks	20–60	upright	**TB18**	2–4 weeks	20–60	horizontal
**TB6**	<2 weeks	20–60	upright	**TB19**	<2 weeks	20–60	upright
**TB7**	2–4 weeks	20–60	upright	**TB20**	2–4 weeks	10–20	upright
**TB8**	4–12 weeks	>60	upright	**TB21**	>12 weeks	20–60	horizontal
**TB9**	4–12 weeks	>60	horizontal	**TB22**	>12 weeks	20–60	horizontal
**TB10**	4–12 weeks	>60	upright	**TB23**	4–12 weeks	20–60	upright
**TB11**	4–12 weeks	>60	upright	**TB24**	4–12 weeks	20–60	upright
**TB12**	4–12 weeks	20–60	upright	**TB25**	<2 weeks	20–60	upright
**TB13**	2–4 weeks	10–20	upright				

**Table 5:** Microbial count of the tested toothbrushes on different culture media. Means with standard error are shown (*n* = 25).

Culture Medium	Microorganisms	Microbial Count (cfu/toothbrush)	Standard Error
TSA (aerobic), 37 °C	aerobic mesophilic bacterial count	7.05 × 10^6^	5.10 × 10^5^
TSA (anaerobic), 37 °C	anaerobic mesophilic bacterial count	1.19 × 10^7^	1.24 × 10^6^
Columbia Blood Agar, 35 °C	*Streptococcus* spp.	3.85 × 10^6^	3.04 × 10^5^
MacConkey, 35 °C	gram-neg. bacteria	5.04 × 10^6^	7.27 × 10^5^
MEA, 30 °C	yeast and fungi	1.42 × 10^6^	1.26 × 10^5^

**Table 6:** Overview of resistance genes detected in toothbrush (TB) samples. IntI1 and bla genes are listed in descending order of the absolute abundance in gene copies mL^−1^.

Sample	Resistance Genes	Sample	Resistance Genes
**TB1**	*intI1, bla*_ACT/MIR_, *bla*_OXA-23_, *bla*_OXA-58_	**TB14**	no genes detected
**TB2**	*intI1, bla*_OXA-23_, *bla*_OXA-58_	**TB15**	*intI1*
**TB3**	*intI1, bla*_OXA-48_	**TB16**	*bla*_KPC_
**TB4**	*intI1, bla*_CMY-2_*, bla*_GES_, *bla*_OXA-23_, *bla*_OXA-58_	**TB17**	*bla*_CMY-2_, *bla*_OXA-23_
**TB5**	*intI1, bla*_GES_, *bla*_OXA-58_	**TB18**	*intI1, bla*_CMY-2_, *bla*_OXA-23_
**TB6**	no genes detected	**TB19**	*intI1*
**TB7**	*bla*_OXA-58_	**TB20**	*intI1, bla*_ACT/MIR_, *bla*_CMY-2_, *bla*_OXA-48_
**TB8**	*intI1, bla*_OXA-23_, *bla*_OXA-58_	**TB21**	*intI1, bla*_GES_
**TB9**	*bla*_OXA-23_, *bla*_OXA-58_	**TB22**	*intI1, bla*_KPC_, *bla*_GES_
**TB10**	*intI1, bla*_OXA-58_	**TB23**	*intI1, bla*_KPC_
**TB11**	*intI1*	**TB24**	*intI1, bla*_GES_
**TB12**	*intI1, bla*_ACT/MIR_*, bla*_GES_, *bla*_OXA-58_	**TB25**	*intI1, bla*_ACT/MIR_, *bla*_CMY-2_, *bla*_OXA-58_
**TB13**	*intI1, bla*_OXA-58_		

## Supplementary Materials

The following are available online at https://www.mdpi.com/2076-2607/8/9/1379/s1, Table S1: Detailed summary of taxa (phylum, class, order, family, and genus) found on toothbrushes.
